# Decoding Neuropsychiatric Lupus: A Systematic Review of Anti-N-Methyl-D-Aspartate and Anti-ribosomal P Antibodies

**DOI:** 10.7759/cureus.111730

**Published:** 2026-06-29

**Authors:** Raghavee Neupane, Mollie Goudy, Pujita Julakanti, Oksana Denis, Brian Ingram, Talia Aftab, Nabiha T Atiquzzaman, Raymond L Ownby, Marc M Kesselman

**Affiliations:** 1 Medicine, Nova Southeastern University Dr. Kiran C. Patel College of Osteopathic Medicine, Fort Lauderdale, USA; 2 Medicine, Nova Southeastern University Dr. Kiran C. Patel College of Osteopathic Medicine, Davie, USA; 3 Psychiatry and Behavioral Sciences, Nova Southeastern University Dr. Kiran C. Patel College of Osteopathic Medicine, Davie, USA; 4 Rheumatology, Nova Southeastern University Dr. Kiran C. Patel College of Osteopathic Medicine, Davie, USA

**Keywords:** anti-double-stranded dna antibodies, anti-ribosomal p antibodies, neuropsychiatric lupus, nmda receptor antibodies, systemic lupus erythematosus

## Abstract

Neuropsychiatric systemic lupus erythematosus (NPSLE) includes a wide range of neurological and psychiatric symptoms and is often difficult to diagnose. Autoantibody-mediated neuronal injury has been proposed as a central pathogenic mechanism in NPSLE, particularly involving anti-N-methyl-D-aspartate (NMDA) (anti-NR2/anti-N-methyl-D-aspartate receptor (NMDAR)) antibodies and anti-ribosomal P antibodies. This systematic review evaluated whether these antibodies predict neuropsychiatric or cognitive manifestations in patients with established SLE and assessed their clinical utility for risk stratification. A systematic search of Embase, Web of Science, and Ovid (MEDLINE) identified English-language studies published between January 2015 and December 2025 examining associations between anti-NR2/anti-NMDAR and/or anti-ribosomal P antibodies and neuropsychiatric or cognitive outcomes in SLE. Eighteen studies met the inclusion criteria following Preferred Reporting Items for Systematic Reviews and Meta-Analyses (PRISMA)-guided screening and quality appraisal. Across cohorts, anti-NR2/anti-NMDAR antibodies were most consistently associated with neuropsychiatric phenotypes but showed no reproducible association with objective cognitive impairment or longitudinal cognitive decline. Anti-ribosomal P antibodies showed stronger associations with psychiatric symptom burden. Similarly, anti-ribosomal P positivity was not reliably linked to isolated global cognitive dysfunction. Overall, current evidence suggests that these antibody profiles assist in phenotypic stratification of NPSLE but cannot serve as a standalone predictive biomarker for cognitive impairment. This underscores the need for a multimodal clinical evaluation.

## Introduction and background

Systemic lupus erythematosus (SLE) is a chronic autoimmune disease characterized by immune-mediated inflammation and multiple-organ damage, most commonly affecting the mucocutaneous, musculoskeletal, hematologic, and renal systems [1]. An estimated 204,000 individuals in the United States and 3.4 million people worldwide are affected by SLE, with disease prevalence and disease burden continuing to rise [1,2], particularly among women and individuals of Asian, Black, Hispanic, and Indigenous descent, who experience higher incidence, severity, and mortality [3].

One of the most clinically challenging complications of SLE is neuropsychiatric SLE (NPSLE). NPSLE encompasses a heterogeneous spectrum of central and peripheral nervous system involvement that contributes significantly to morbidity, diagnostic uncertainty, and long-term disability. Neurological and psychiatric manifestations occur in approximately 20-40% of patients during the disease course, sometimes presenting early [4-7]. NPSLE presents a wide range of clinical presentations, from nonspecific symptoms, such as headache, cognitive dysfunction, and mood disturbances, to severe manifestations, including seizures, stroke, psychosis, and encephalitic syndromes [1]. This heterogeneity reflects multiple pathogenic mechanisms, including neuroinflammatory, ischemic, and antibody-mediated processes, and contributes to significant diagnostic and management challenges. Individuals with NPSLE experience a reduced quality of life compared to those without neuropsychiatric involvement, underscoring the need for better mechanistic understanding and earlier identification of at-risk patients [8].

Emerging evidence suggests specific autoantibody profiles may correlate with distinct neuropsychiatric phenotypes in SLE. Autoantibody clustering studies indicate patients with high titers of anti-double-stranded DNA (anti-dsDNA), anti-nucleosome, and anti-ribosomal P antibodies often present with more complex disease forms, including higher frequencies of CNS manifestations such as seizures, psychosis, and headache [9]. The presence of multiple autoantibodies in most patients highlights the profound immunologic heterogeneity of SLE and suggests that cumulative autoantibody burden may contribute to the complexity of neuropsychiatric disease [10,11].

Among these candidate antibodies, anti-ribosomal P antibodies have been extensively studied in the context of neuropsychiatric involvement of SLE. Anti-ribosomal P antibodies target ribosomal phosphoproteins (P0, P1, and P2) and have long been implicated in the pathogenesis of NPSLE [12]. These antibodies are found in approximately 10-20% of patients with SLE, with a higher prevalence in those with neuropsychiatric involvement, particularly those presenting with manifestations like lupus psychosis and mood disorders [8,13]. Mechanistically, anti-ribosomal P antibodies are thought to bind neuronal surface proteins and alter neurotransmission, potentially contributing to affective and behavioral symptoms [10]. Clinically, anti-ribosomal P antibody positivity has been associated with psychosis, depression, anxiety, and higher global disease activity scores in several cohorts. In patients with NPSLE, it has been linked to a worse prognosis [13,14]. However, findings across studies remain inconsistent. Anti-ribosomal P antibodies do not reliably distinguish NPSLE from non-NPSLE, nor do they consistently predict future neuropsychiatric disease or cognitive decline in asymptomatic patients [8,14]. Thus, their clinical utility appears context-dependent and insufficient to support their use as a standalone predictive biomarker.

One proposed mechanism linking autoimmunity to neuronal injury in SLE involves a subset of anti-dsDNA antibodies that cross-react with the NR2 subunit of N-methyl-D-aspartate (NMDA) receptor. Under conditions of systemic and/or CNS inflammation, characterized clinically by active SLE manifestations and paraclinically by elevated anti-dsDNA titers, hypocomplementemia, and increased inflammatory markers such as EST and CRP, blood-brain barrier integrity may be compromised. This disruption permits circulating anti-NR2 antibodies to access the CNS and bind neuronal NMDA receptors. Such binding can induce receptor dysfunction, excitotoxicity, and neuronal damage, contributing to the development of neuropsychiatric manifestations [10,11]. Anti-NMDA receptor (anti-NR2) antibodies are detected in approximately 25-40% of patients with SLE and in up to 60% of patients with NPSLE. Higher levels have been reported in patients with active neuropsychiatric involvement compared to those with focal or non-inflammatory CNS disease [8,13,15].

Although serum levels of anti-dsDNA and anti-NMDA receptor antibodies do not consistently correlate with neuropsychiatric activity, higher antibody titers have been reported in patients with active NPSLE [8]. Despite strong experimental and clinical evidence supporting their pathogenic role, the utility of anti-NMDA receptor antibodies as predictive biomarkers remains uncertain. Studies of serum and cerebrospinal fluid have demonstrated associations between antibody presence and neuropsychiatric symptoms. However, antibody titers do not consistently correlate with disease activity. In addition, variability in assay methodologies further limits their reliability as standalone early diagnostic or prognostic tools. These inconsistencies highlight a critical gap in current knowledge regarding whether NMDA receptor antibodies or anti-ribosomal P antibodies precede, predict, or merely accompany neuropsychiatric manifestations in patients with established SLE [13,15].

The purpose of this review was to evaluate whether anti-NMDA receptor (NR2) antibodies and anti-ribosomal P antibodies in patients with long-standing SLE predict the development of cognitive or neuropsychiatric symptoms and explore the implications for surveillance strategies and early clinical intervention.

## Review

Methods

A comprehensive literature search was conducted using the Embase, Web of Science, and Ovid (MEDLINE) databases. Search terms included keywords and controlled vocabulary related to SLE (e.g., "SLE", "lupus", "Systemic lupus erythematosus"), AND NMDA-R/NR2 antibodies (e.g., "NMDA receptor", "NR2 receptor", "anti-NR2", "anti-GluN2", “anti-NMDA”, “anti-NMDAR antibody”) OR ("anti-ribosomal P" OR "ribosomal P antibody" OR "anti-ribosomal P protein antibody" OR "anti-Rib-P" OR "anti-RP" OR "anti-P antibody"). These terms were combined with neuropsychiatric and cognitive outcome terms (e.g., "neuropsychiatric lupus", "NPSLE", OR "cognitive dysfunction" OR "cognitive impairment" OR "mood disorder" OR "psychosis" OR "seizure" OR "neurocognitive disorder". Boolean operators ("AND" and "OR") were used to combine search terms appropriately.

To ensure article recency, only English-language articles published between January 1, 2015, and December 1, 2025, were assessed. Titles and abstracts were screened using the Rayyan systematic review platform [16], which facilitated duplicate removal and a two-tier review (initial title/abstract screening followed by full-text evaluation), performed by independent human reviewers (RN, MG, PJ, TA, BI). The search was limited to peer-reviewed, published studies. The Nova Southeastern University (NSU) library database was utilized to access databases and full-text articles.

Eligibility Criteria

The population of interest included adult non-pregnant patients with SLE, including those with suspected or confirmed NPSLE. The exposures of interest were the presence of NMDA receptor-related antibodies, including anti-NR2 (anti-GluN2, anti-NMDA, or anti-NMDAR) antibodies, as well as anti-ribosomal P antibodies. When available, comparator groups included patients with SLE without neuropsychiatric manifestations, individuals with other autoimmune or neurological conditions, and healthy controls. The primary outcomes of interest were the diagnostic association and performance of these antibodies in identifying neuropsychiatric or cognitive manifestations of SLE, including measures such as sensitivity, specificity, and overall diagnostic accuracy.

Studies were considered eligible if they evaluated NMDA receptor-related antibodies or anti-ribosomal P antibodies in patients with SLE and assessed their role in the diagnosis or identification of neuropsychiatric or cognitive involvement. Eligible study designs included clinical trials, observational studies (cohort, case-control, or cross-sectional), and relevant human translational studies.

Studies were excluded if they examined these antibodies in contexts unrelated to SLE, such as primary psychiatric disorders, non-SLE autoimmune diseases, oncology, or infectious etiologies. Articles focusing solely on treatment response, prognosis, or mechanistic pathways without diagnostic relevance were also excluded. Reviews, editorials, commentaries, conference abstracts, animal-only studies, in vitro experiments, non-English publications, and studies without full-text availability were excluded.

Study Selection and Critical Appraisal of the Evidence

The initial search yielded 267 studies. After removing 105 duplicates, 162 unique articles remained. Of these, 20 were the wrong study design, including 10 case reports, 7 literature reviews, 2 were both (case reports and literature reviews), and 1 systematic review/meta-analysis: 28 were outdated; 59 focused on the wrong population, including animals, children, and pregnant women; and 23 studies focused on research unrelated to the topic. The remaining 32 studies were screened through a blinded, two-tiered review process conducted by two independent reviewers, with a third reviewer resolving any discrepancies.

Quality assessment was performed using the Joanna Briggs Institute Critical Appraisal Tools [17], which categorized risk of bias as low (> 70%), moderate (50-70%), or high (< 50%) [18]. Following this assessment, all 29 articles that met the inclusion criteria were included in the final analysis.

**Figure 1:**
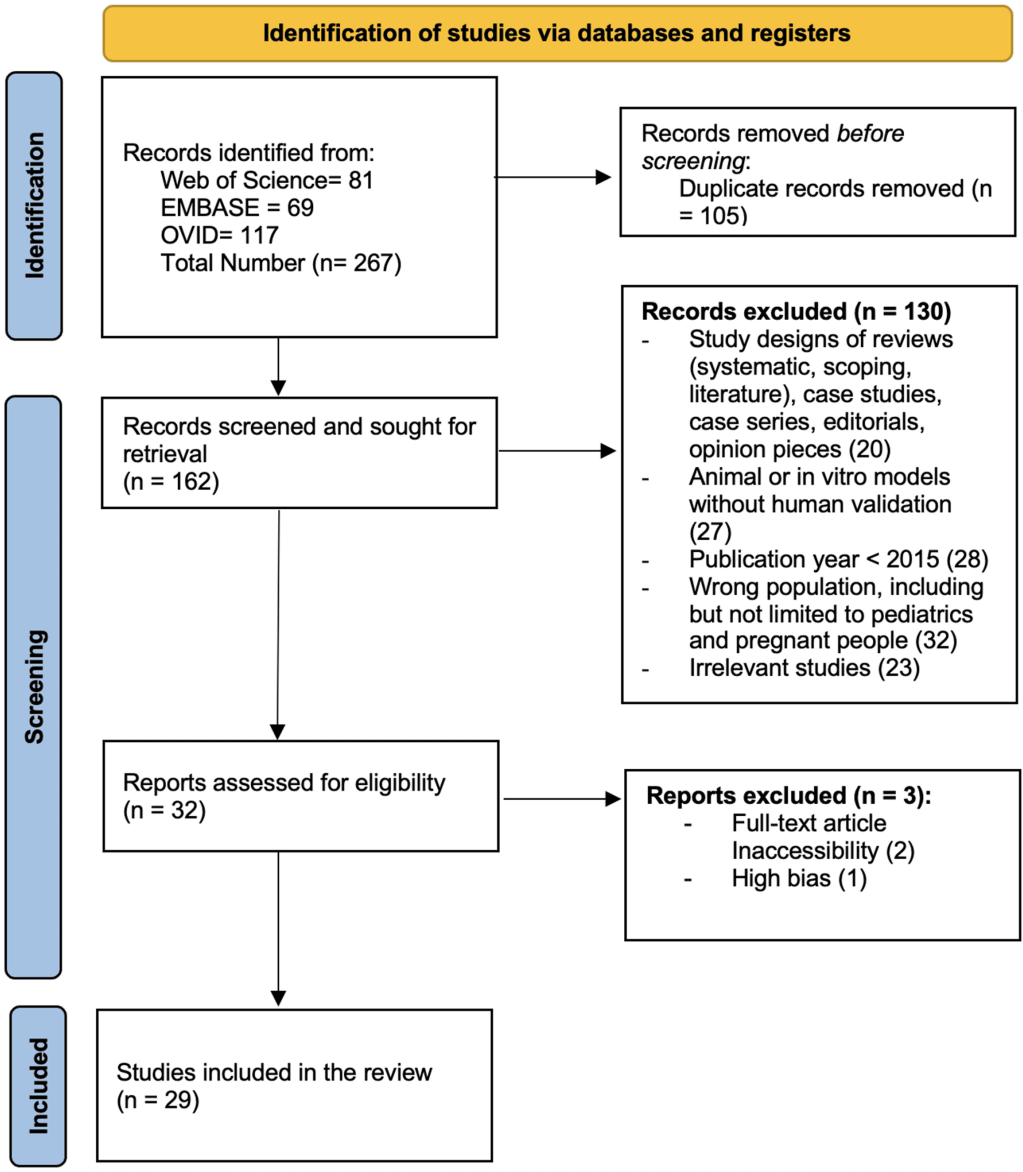
Preferred Reporting Items for Systematic Reviews and Meta-Analyses (PRISMA) flow diagram illustrating the study selection process.

The review process adhered to Preferred Reporting Items for Systematic Reviews and Meta-Analyses (PRISMA) guidelines, and a PRISMA flow diagram was developed to illustrate the article selection process (Figure 1).

Results

The included studies evaluated the relationships between NMDA receptor-related antibodies, anti-ribosomal P antibodies, and neuropsychiatric manifestations in SLE patients. Study designs, patient populations, and outcome measures varied across investigations; however, collectively, the literature provided insight into how these antibodies relate to neuropsychiatric symptoms, disease manifestations, fatigue, and cognitive outcomes.

Across multiple studies, serum anti-NR2/anti-NMDAR antibodies have been reported to correlate with specific neuropsychiatric manifestations, such as mood disorders, psychosis, and seizures. These associations are primarily observed as higher antibody levels in patients with neuropsychiatric symptoms compared with SLE patients without such manifestations. Although effect sizes and the strength of correlation vary across cohorts, the overall pattern consistently supports a link between elevated anti-NR2/anti-NMDAR antibodies and neuropsychiatric features. Elevated levels of anti-NR2A and anti-NR2B antibodies were observed, particularly among patients with seizure disorders and fibromyalgia [19,20]. Anti-NR2 antibodies were also more frequently detected in active NPSLE and correlated with higher disease activity indices [21]. Neuropsychiatric presentations, including acute confusional state and mood disorders, have also been linked to anti-DNA and anti-NR2 antibodies [22]. Additionally, anti-NR2 positivity was associated with fatigue severity, particularly general fatigue rather than cognitive fatigue [23,24].

Serum anti-NR2 antibodies were not associated with a change in performance on standardized neuropsychological testing in patients with long-standing SLE, including domains assessed by standardized cognitive batteries [25]. Similarly, anti-NMDA levels were not correlated with Montreal Cognitive Assessment (MoCA) scores in cross-sectional analysis [26]. Longitudinal data further demonstrated that anti-NMDAR antibodies did not predict cognitive decline or accrual of damage over time [27]. Collectively, available evidence suggested anti-NR2/anti-NMDAR antibodies were more closely related to specific neuropsychiatric symptoms (seizures, acute confusional states, fibromyalgia, and fatigue) than to consistent global cognitive dysfunction across SLE populations.

Anti-NR2 antibodies demonstrated stronger associations with neuropsychiatric symptoms and fatigue, mainly reflected in higher antibody levels in affected patients. Other autoantibodies show distinct but related patterns: for example, anti-ribosomal P antibodies are more commonly found in patients with psychiatric symptoms and higher overall disease severity. This suggests a stronger connection to psychiatric symptoms burden and overall disease severity than with anti-NR2 antibodies.

Anti-ribosomal P antibodies demonstrated stronger and more consistent associations with psychiatric manifestations and overall disease severity than with isolated cognitive impairment. Multiple cohorts reported higher anti-ribosomal P positivity among patients with NPSLE, with significant associations observed for epilepsy, stroke, and broader neuropsychiatric involvement [28,29]. Psychiatric symptom burden, particularly the severity of depression and anxiety, was repeatedly associated with anti-P positivity across independent populations [30-32]. Beyond symptom association, anti-ribosomal P positivity was linked to greater disease activity and more severe clinical phenotypes, including higher Systemic Lupus Erythematosus Disease Activity Index-2000 (SLEDAI-2K) scores and increased early and long-term mortality in NPSLE [33,34].

However, evidence supporting a direct relationship with cognitive impairment was limited. Anti-ribosomal P positivity was not associated with cognitive dysfunction or abnormalities in event-related potentials among patients with long-standing SLE [14]. Similarly, another cross-sectional cohort found no significant association between anti-ribosomal P and overall NPSLE status or major organ involvement [35]. Overall, the available literature supported a stronger relationship between anti-ribosomal P antibodies and psychiatric symptom burden and disease severity than with reproducible, standalone cognitive impairment. Table 1 summarizes the included studies, detailing the authors/year, study design, Psychiatric Presentation, Biomarkers (antibodies), Key Findings, Limitations, and Risk of Bias (JBI).

**Table 1:** Summary of study characteristics, findings, and risk-of-bias assessment using the JBI Critical Appraisal Tools. ACS = acute confusional state; AIDP = acute inflammatory demyelinating polyneuropathy; ANAM4 = Automated Neuropsychological Assessment Metrics, Version 4; ANNA = anti-neuronal nuclear antibodies; anti-dsDNA = anti-double-stranded DNA antibody; anti-GluN2B = antibody to glutamate ionotropic NMDA receptor subunit 2B; anti-NMDAR = anti-N-methyl-D-aspartate receptor antibody; anti-NR1/2/2A = antibody to NMDA receptor subunit 1/2/2A; anti-RNP/anti-U1RNP = anti-U1 ribonucleoprotein antibody; β2GPI = beta-2 glycoprotein I; ERP = event-related potentials; FGF = fibroblast growth factor; G-CSF = granulocyte colony-stimulating factor; GABAR = gamma-aminobutyric acid receptor; GM-CSF = granulocyte-macrophage colony-stimulating factor; GluN2B = glutamate ionotropic receptor NMDA type subunit 2B; ICAM-1 = intercellular adhesion molecule-1; IFN-α/γ = interferon alpha/gamma; IL-1β = interleukin-1 beta; IL-1ra = interleukin-1 receptor antagonist; IL = interleukin; IP-10 = interferon gamma-induced protein 10 (CXCL10); LA = lupus anticoagulant; MCP-1 = monocyte chemoattractant protein-1; MIG = monokine induced by gamma interferon (CXCL9); MIP-1α = macrophage inflammatory protein-1 alpha; MIP-1β = macrophage inflammatory protein-1 beta; MOG = myelin oligodendrocyte glycoprotein; NMDAR = N-methyl-D-aspartate receptor; NfL = neurofilament light chain; NPSLE = neuropsychiatric SLE; NR = NMDA receptor subunit; PAI-1 = plasminogen activator inhibitor-1; PDGF = platelet-derived growth factor; PHQ-9 = Patient Health Questionnaire-9; PSNP-SLE = post-steroid neuropsychiatric SLE; RANTES = regulated upon activation, normal T cell expressed and secreted; S100B = S100 calcium-binding protein B; SLEDAI = SLE Disease Activity Index; SRT = simple reaction time; SRRS = Social Readjustment Rating Scale; SS = Sjögren’s syndrome; TNF-α = tumor necrosis factor alpha; TWEAK = TNF-like weak inducer of apoptosis; VEGF = vascular endothelial growth factor; WSAS = Work and Social Adjustment Scale

Authors, Year	Study Design	Patients (M/F)	Mean Age (years)	Mean Disease Duration (years)	Psychiatric Presentation	Biomarkers (antibodies)	Key Findings	Limitations	Additional Findings	Conclusion	Risk of Bias (JBI)
Talukdar et al. (2020) [9]	Cross-sectional	145 SLE	25.89 +/- 8.13	Not stated	Seizure, psychosis, headache	Anti-P	Patients with a combination of high titers of anti-dsDNA, anti-nucleosome, and anti-P had higher frequencies of CNS manifestations	Hospital-based with a small sample size	Neutrophil:lymphocyte ratio was higher in SLE patients with renal vs non-renal complications	Patients with increased anti-dsDNA, anti-nucleosome, and anti-P associated with increased CNS manifestations of SLE	Low
Szmyrka et al. 2019 [14]	Cross-sectional observational study	SLE (2M/35F): 37, NPSLE: 24, non-NPSLE: 13	38.3	10	Cognitive impairment (memory, attention/executive function, visuospatial function), mood disorders (mainly depression), cerebrovascular disease (stroke, TIA, sinus thrombosis), seizures, psychosis, polyneuropathy, cranial neuropathy, acute confusional state, severe headache	Anti-P	Anti-P positivity did not differ between SLE patients with and without cognitive impairment and was not associated with neuropsychological deficits	Small sample size (n=37). No longitudinal follow-up; depression is a confounder	Event-related potential abnormalities suggest subclinical dysfunction but correlate less strongly with clinical or immunologic measures than neuropsychological testing, while select autoAbs (e.g., anti-dsDNA, aCL) associate with deficits in specific cognitive domains	Serum anti-P Abs not associated with cognitive impairment or ERP abnormalities and therefore do not function as early biomarkers of emerging neuropsychiatric symptoms	Low
Park et al. 2017 [20]	Case-control study	214 (205F, 9M)	SLE: 34, Control: 36.5	8	CNS: acute confusional state, psychosis, cerebral infarction, demyelinating syndrome, aseptic meningitis, seizure disorder, cognitive dysfunction. PNS: polyneuropathy. Psychiatric: affective disorders (depression and bipolar disorder).	Anti-NMDAR (anti-GluN2B)	Anti-GluN2B antibody titers higher in SLE patients compared to fibromyalgia-only patients and healthy controls; antibody positivity independently predicted both concomitant fibromyalgia (OR ~4.18) and neuropsychiatric SLE (OR ~4.36), even after adjustment for age, sex, and disease activity	Small antibody-positive sample size; weak correlations with pain severity; limited generalizability due to a single-ethnic cohort	Anti-GluN2B positivity associated with higher widespread pain index scores and lower C4 levels, but not with anti-dsDNA titers, lupus nephritis, affective disorders, or fibromyalgia without SLE	Serum anti-NMDAR (GluN2B/NR2) positivity in long-standing SLE associated with fibromyalgia and neuropsychiatric manifestations, supporting its role as a potential early biomarker of CNS involvement even without CSF testing	Low
Hirohata et al. (2019) [21]	Cross-sectional observational study	31 NPSLE (22 diffuse NPSLE, 9 focal NPSLE)	Not stated	Not stated	Diffuse: psychiatric/neuropsychological syndromes (17 with acute confusional state). Focal: neurological syndromes or peripheral nervous system involvement.	Anti-NR1/NR2 Anti-P	Serum anti-NR2 Abs common in diffuse NPSLE, particularly diffuse disease, and correlate with disease activity. Anti-NR1/NR2 Abs were rare, indicating classical anti-NMDAR encephalitis mechanisms are uncommon in SLE	Small sample size; sex, age, and disease duration not reported; no longitudinal follow-up; confounding factors (e.g., medications, disease activity) not adjusted for	Anti-NR2 correlates with anti-Sm Abs (suggesting generalized abnormal B-cell responses rather than antigen-specific)	Although serum anti-NR2 and anti-P elevated in diffuse NPSLE, there is no evidence that anti-NR2 predicts emerging cognitive/psychiatric symptoms in CSF-negative SLE patients	Low
Fujieda et al. 2019 [22]	Retrospective observational cohort study	49 patients with NPSLE De novo NPSLE: 24 (M/F: 3/21) PSNP-SLE: 25 (M/F: 4/21)	Median at onset: de novo NPSLE 26, PSNP-SLE 22 Median on admission: de novo NPSLE 33, PSNP-SLE 36	De novo NPSLE: median 2.42 PSNP-SLE: median 1.92	Diffuse: Acute confusional state. Mood disorder: psychosis. Cognitive dysfunction: anxiety disorder. Focal: CNS, PNS, cerebrovascular disease	Anti-DNA/NR2	Anti-DNA/NR2 Abs present at high titers in both de novo NPSLE and PSNP-SLE; Diffuse neuropsychiatric manifestations (ACS, mood disorders) more frequent in PSNP-SLE, and focal manifestations (cerebrovascular disease) predominated in de novo NPSLE, supporting anti-DNA/NR2 Abs as a potential predictive factor for PSNP-SLE	Small sample size (n=49)	Anti-DNA/NR2 Abs associated with diffuse manifestations, supporting pathogenic role in neuronal injury via NMDA receptor binding and blood-brain barrier disruption	Anti-DNA/NR2 Abs elevated in both de novo NPSLE and PSNP-SLE, with a strong anti-dsDNA correlation in PSNP-SLE, and are associated with diffuse neuropsychiatric manifestations, suggesting anti-DNA/NR2 titers are a potential predictive value for PSNP-SLE pending prospective validation	Low
Schwarting et al. (2019) [23]	Cross-Sectional study with experimental and longitudinal components	426 patients, treatment effects studied in 86	Range: 18-76	Not stated	Severity of fatigue + depression correlations	Anti-NSR2	Anti-NR2 showed a strong correlation between fatigue severity and disease activity	Heterogeneity among the groups, self-reported symptoms	Anti-NR2 is a useful biomarker for fatigue and may guide therapy in SLE	Anti-NR2 is a useful biomarker for fatigue and may guide therapy in SLE	Low
Marinoska et al. (2023) [24]	Observational cross-sectional study	88 patients, 32 with SLE	50.97	Not stated	Cognitive fatigue	Anti-NR2	Anti-NR2 correlation between cognitive fatigue in some autoimmune diseases but not in SLE; anti-NR2 is correlated with non-cognitive fatigue in patients with SLE, SS, and other autoimmune rheumatic diseases	Anti-NR2 titers only classified as positive or negative; small sample size; no CSF/imaging obtained	Anti-NR2 elevated in autoinflammatory rheumatic diseases; No correlation with fatigue severity and anti-NR2 titers	No significant association between elevated anti-NR2 titers and cognitive fatigue in patients with SLE; however, there is an association between anti-NR2 levels and general fatigue	Low
Gulati et al. 2016 [25]	Cross-sectional observational study	57 (4M/53F)	SLE: 49.9	13.1	Cognitive dysfunction --> measured by ANAM4 includes SRT and eight subtests that measure short-term and long-term recall, learning, working memory, sustained attention, logical reasoning, and mathematical and visual-spatial processing	Serum anti-NR2	Serum Anti-NR2 antibody levels were not associated with cognitive dysfunction, even after adjustment for demographic and clinical confounders	Small cognitively impaired sample; limited sensitivity of BBB biomarkers for transient or prior disruption; potential temporal mismatch between cognitive dysfunction and biomarker sampling	Cognitive performance was independently associated with age, ethnicity, opioid use, and reaction time, not immunologic markers; serum S100B and anti-S100B were not independently linked to cognition, supporting the hypothesis that pathogenic anti-NR2 antibodies may be intrathecally produced	Serum anti-NR2 positivity did not correlate with cognitive dysfunction in long-standing SLE, arguing against its use as a standalone early biomarker without supportive CSF findings	Low
Suntoko et al. (2023) [26]	Cross-sectional	56 SLE	34 +/- 9	3.83 +/- 3.5	Visuospatial, naming, attention, language, abstraction, delayed recall, orientation	Anti-NMDA	Anti-NMDA levels were not correlated with cognitive dysfunction	Small sample size	IL-6 levels have a weak negative correlation with cognitive dysfunction; young patients had more severe cognitive dysfunction regarding delayed recall and orientation, as well as increased IFN-α levels	There is no correlation between anti-NMDA and cognitive dysfunction measured by the MOCA; however, higher IL-6 levels were associated with decreased MOCA scores	Low
Mimica et al. (2019) [27]	Observational cross-sectional	SLE: 99	Not stated	Not stated	Major depression, cognitive deficit	Anti-NMDAR, anti-P	Increased anti-P and anti-NMDAR are not associated with accrual damage	Only female patients; short follow-up; small subgroup sizes	Accrual damage is not associated with depression or suicide risk in SLE patients; anti-NPSA levels could potentially be linked to accrual damage	Anti-P and anti-NMDAR levels are not able to predict the incidence of depression or cognitive deficits	Low
Taha et al. 2022 [28]	Cross-sectional, case-control study	SLE: 90, NPSLE: 30, non-NPSLE: 60	NPSLE: 36.6, non-NPSLE: 37.3	SLE: 4.7, NPSLE: 3.4	CNS: Epilepsy (30%), stroke (26.7%), headache (13.3%), confusion (53.3%), demyelinating syndromes, cranial nerve palsy, and cognitive impairment (40% borderline MMSE scores). Psychiatric: anxiety (46.7%), mood disorders, psychosis	Serum anti-P	Anti-P positivity was significantly higher in NPSLE patients compared with non-NPSLE SLE controls (46.7% vs 20%, P=0.001), and showed a strong association with neuropsychiatric manifestations (epilepsy and stroke).	Single-center, no longitudinal follow-up, small sample size, and limited generalizability beyond the Sudanese population.	NPSLE patients had higher ANA positivity and increased anti-histone, anti-nucleosome, and antiphospholipid antibodies. Neurological manifestations correlated most strongly with ANA positivity and were accompanied by anemia, leukocytosis, thrombocytopenia, and hypoalbuminemia.	Anti-P Abs are strongly associated with NPSLE, particularly early CNS involvement, supporting diagnostic utility, but evidence is insufficient to justify surveillance escalation or immunosuppressive intensification in asymptomatic patients without longitudinal data.	Low
Zhang et al. 2021 [29]	Retrospective observational cohort study	SLE: 194 (14M/180F) NPSLE: 194	29.8	SLE 3.09, NPSLE 2.78	CNS: Seizure, acute confusional state, cerebrovascular disease, headache, psychosis, cognitive impairment, mood disorder, demyelination, dyskinesia, myelitis, aseptic meningitis, anxiety disorder. PNS: cranial neuropathy, AIDP. Single/multiplex mononeuropathy, polyneuropathy	Anti‑P	NPSLE patients had more frequent anti-P positivity	Small control group; restriction to hospitalized NPSLE patients; short follow-up; single-center setting	Elevated SLEDAI‑2K and hypocomplementemia predicted shorter survival	High disease activity and anti-P positivity were associated with neuropsychiatric involvement and poorer survival, suggesting that anti-P reflects NPSLE risk and severity	Low
Duca et al. 2023 [30]	Cross‑sectional observational clinical study	65 SLE	Not stated	At least 6 months, not explicitly stated	Depression: severe (12.3%), moderate (33.8%), mild (40%). Anxiety: prevalent in 98.5% (mild-very severe)	Serum anti-P	Anti-P positivity (43.1%) was associated with greater depression and anxiety severity in SLE. In linear regression, anti-P and PAI-1 predicted depression	Absence of baseline NPSLE diagnoses with outcomes limited to symptom severity rather than clinical classification; enrollment restricted to inactive disease, limiting applicability to active NPSLE	Higher depression and anxiety scores were associated with increased anti-β2GPI, lupus anticoagulant, and ICAM-1 levels, while lower complement C3/C4 correlated with worse symptoms	Anti‑P Abs were associated with greater severity of depression and anxiety, suggesting autoimmune/inflammatory processes contribute to psychiatric symptom burden	Low
Pradhan et al. (2015) [31]	Observational, cross-sectional, double-blinded study	NPSLE: 60, non-NPSLE: 60	NPSLE: 23 +/- 9.5, non NPSLE: 26 +/- 10	Not stated	Mood disorder	Anti-P	Mood disorders were significantly associated with Anti-P; however, there was no significant difference between Anti-P levels in NPSLE vs non-NPSLE	Small sample size	ANNA is not associated with neurological symptoms of SLE	Neither anti-P nor ANNA is significantly elevated in non-NPSLE vs NPSLE patients	Low
Chessa et al. (2023) [32]	Cross-sectional, case-control study	SLE: 33 (3M, 30F)	43.5	10.4	Depressive symptoms + cognitive performance	Anti-P, anti-NR2	Anti-P positive in 6 patients, anti-NR2 positive in 14 patients. Depressive symptoms were found in 14 patients, and 3 patients were positive for both. 19 patients showed cognitive changes that were not associated with autoAbs. Serum anti-P did show a correlation with depressive episodes in 14 patients. Serum anti-P levels have an association with changes seen on fMRI; however, anti-NR2 and daily prednisone use did not have an effect	Did not check for autoAbs in CSF; small sample size; study design did not allow for long-term outcomes; hard to determine the correlation between serum anti-P vs secondary factors. Duration of disease affecting cognition and compensatory mechanisms of the brain. Unable to determine the effects of alternative treatments for depressive episodes/cognitive dysfunction	57.6% had cognitive impairment; prednisone dose also linked to depression	Anti-P antibodies may contribute to depression via brain network disruption in SLE	Low
Ma et al. 2025 [33]	Single-center, retrospective observational study	SLE: 388 (39M/349F)	Median: 35	Median: 1	CNS lupus, depression, and psychosis	Anti-P	Anti-P-positive SLE was characterized by higher disease activity (SLEDAI-2K 15 vs 12), and neurologic involvement was more common but did not reach statistical significance due to limited sample size	Single-center; retrospective design with potential selection bias and missing data; a prolonged study period with evolving laboratory methods; Absence of treatment or longitudinal follow-up analyses	Anti-P Abs may contribute to SLE pathogenesis through pro-inflammatory cytokine induction (IL-6, TNF-α), direct neuronal and hepatocellular effects, and potential anti-lymphocyte activity on activated T cells	Anti-P positive SLE is characterized by higher disease activity, though neurologic associations were not statistically significant	Low
Arinuma et al. 2019 [34]	Retrospective observational cohort study	55 diffuse NPSLE (9M/46 F)	34	Median: 1 month	Acute confusional state, psychosis, mood disorder, cognitive dysfunction, anxiety disorder	Serum Anti-P	Anti-P Abs were present in 41.8% of diffuse NPSLE and were significantly more frequent in fatal cases of diffuse NPSLE. Positivity independently predicted increased overall mortality (RR 2.68), shorter survival (HR 5.24), and higher early (<1-year) mortality after NPSLE onset	Small sample size; no longitudinal anti-P measurements	Anti-P positivity was associated with major non-renal organ damage but not with specific psychiatric subtypes (ACS, psychosis, mood disorder, cognition). Anti-DNA positivity was protective against early mortality, while anti-PL may confer an additive risk in diffuse NPSLE. The authors hypothesize that anti-P contributes via direct neuronal injury, systemic inflammation, BBB disruption, and a refractory SLE phenotype	In diffuse NPSLE, serum anti-P positivity independently predicts early and long-term mortality, reflecting a more severe, refractory disease phenotype	Low
Marín et al. 2021 [35]	Cross‑sectional observational study	SLE: 66 (3M/63F) NPSLE: 12	29	Not stated	Peripheral neuropathy, psychosis, headache, CVA, seizures, organic brain syndrome	Serum anti-P	Anti‑P positivity (25.76%) was not associated with NPSLE, lupus nephritis, or hepatic involvement (p > 0.05)	Causal inference; small sample size	High anti‑P positivity co‑occurred with other autoantibody positivity, suggesting clustering with established SLE serological markers (anti‑Smith, anti‑Ro60/SSA, anti‑dsDNA). A trend toward hypocomplementemia was observed in anti-P-positive subjects	Serum anti-ribosomal P Abs were not associated with NPSLE or other major organ involvement but clustered with anti-Smith, anti-Ro60/SSA, and anti-dsDNA	Low
Hirohata et al. (2021) [36]	Cross-sectional	101 NPSLE: 69 diffuse NPSLE, 32 psychiatric manifestations other than diffuse NPSLE	39 +/- 14	Not stated	Acute confusional state	Anti-NR2, anti-P	Anti-NR2 was elevated in the acute confusional state compared with focal NPSLE; anti-P and anti-NR2 do not correlate with the increase of albumin in the acute confusional state	Anti-sm, anti-NR2, and anti-P only accounted for 10% of the increase in albumin levels; they did not investigate how anti-sm caused damage to the BBB	Albumin was significantly higher in the acute confusional state in diffuse NPSLE vs non-acute confusional state pts due to anti-sm; anti-sm was elevated in the acute confusional state compared to the other groups	Anti-sm is a prognostic factor for acute confusional state and BBB damage in patients with NPSLE, whereas anti-NR2 and anti-P are not; anti-NR2 is a potential marker for acute confusional state in NPSLE	Low
Kamstrup et al. 2025 [37]	Cross-sectional observational study	198 SLE (27M/171F)	47.3	Not stated	NPSLE present in 43 • CNS symptoms in 32 • PNS symptoms in 7	Serum Anti-P	Fourteen patients were anti-P positive; only 3 (21%) had NPSLE, with no significant association between anti-P positivity and NPSLE status; anti-P positivity was significantly associated with higher SLEDAI-2K scores	Unstandardized disease duration; variable timing of NPSLE definition relative to sampling	Anti-P more frequently co-occurred with ANA, anti-dsDNA, anti-histone, and anti-RNP positivity; while logistic regression linked NPSLE associated with disease activity and organ damage, but not to anti-P status	Anti-P Abs were not significantly associated with NPSLE but correlated with higher disease activity, arguing against their use as are not an early predictive biomarker for neuropsychiatric involvement in long-standing SLE without supportive CSF or longitudinal data	Low
Ogawa et al. 2016 [38]	Observational cohort, cross-sectional study	SLE 68, diffuse NPSLE: 22 (1M/21F), focal NPSLE: 19 (1M/18F), non-CNS SLE: 27 (6M/21F), non-SLE rheumatic diseases: 21 (11M/10F)	Diffuse NPSLE: 39.1, focal NPSLE: 38.2, non-CNS SLE: 37.7, non-SLERD: 51.9	Not stated	Diffuse NPSLE: acute confusional state (13), cognitive dysfunction (4), mood disorder (3), psychosis (2). Focal NPSLE: aseptic meningitis (7), cerebrovascular disease (4), seizure disorder (3), demyelinating syndrome (2), polyneuropathy (2), headache (1)	Serum + CSF anti-NR1, anti-NR2 also measured for correlation	Serum anti-NR1, NR1-A, and NR1-C elevated in NPSLE vs non-SLERD. CSF anti-NR1-A and NR1-C significantly elevated in diffuse NPSLE vs focal	Small sample size; cross-sectional design; disease duration not reported; potential cross-reactivity with anti-NR2 and anti-DNA not fully excluded	Elevation of CSF anti-NR1-A/NR1-C may be related to BBB dysfunction, purified anti-NR1-A/NR1-C bind neurons, and distinct epitopes may influence NMDA receptor expression via DREAM interaction	AutoAbs against the NR1 subunit are present in diffuse NPSLE and likely play a role in its pathogenesis	Low
Kondo-Ishikawa et al. 2020 [39]	Retrospective observational cohort study	69 NPSLE (9M/60F) 13 control	34.9	5	Diffuse NPSLE symptoms: acute confusional state: 10.3%. Anxiety disorder: 3.4%. Cognitive dysfunction: 12.6%. Mood disorder: 12.6%. Psychosis: 9.2%. Focal NPSLE symptoms: aseptic meningitis: 2.3%. Cerebrovascular disease: 8%. Demyelinating syndrome: 5.7%. Headache: 21.8%. Chorea: 1.1%. Myelopathy: 2.3%. Seizure disorder: 10.3%	CSF anti-NR2, anti-P, serum anti-NR2	Anti-NR2 Abs were associated with diffuse NPSLE, particularly acute confusional state and lupus psychosis	Small sample size; limited assessment of inflammatory mediators without functional activity testing; lack of statistically significant combined autoantibody effects	Anti-NR2 Ab associated primarily with IL-6/MI, anti-U1RNP with IL-8/MIG; no synergistic effect of anti-NR2 and anti-U1RNP Abs on NPSLE clinical activity	In NPSLE, CSF anti-NR2 and anti-U1RNP Abs preferentially drive distinct inflammatory pathways, anti-NR2 elevate IL-6 and MIG, and anti-U1RNP elevate IL-8 and MIG; double positivity had no additive clinical or SLEDAI effects	Low
Liang et al. (2025) [40]	Case control	47 SLE patients (4 M, 43 F), 33 controls (4 M, 29 F)	SLE group: 37.1 +/- 13.7, Control group: 38.4 +/- 10.7	Not stated	Brain injury	Anti-P, immunoglobulins, anti-phospholipid	SLE patients showed significant changes on resting-state fMRI, including amplitude if low frequency fluctuations and degree of centrality located in the frontal and occipital regions. Those who were anti-P positive showed increased values in areas related to cognitive and emotional regulation	Small sample size. No standardized psychiatric assessment; cross-sectional (no causality); single-center, selection bias, imaging modality variability	Anti-cardiolipin antibody IgM correlates with brain injury in those who are positive for Anti-P antibody.	Neuroimaging can be utilized as an early biomarker to assess brain injury in patients with SLE. ARPA combined with additional immune factors, may play a role in the impairment of certain brain functions	Moderate
Yoshio et al. (2016) [41]	Cross-sectional	30 central NPSLE, 22 non-central NPSLE	37	Not Stated	Confusion, anxiety, psychosis, mood disorder, aseptic meningitis, cerebrovascular disease, myelopathy, seizure	Anti-P	A positive correlation between anti-P titers in the serum vs CSF	Small sample size	No correlation between the cytokines in the serum vs CSF; IL-6 had the most significant increase in the CSF of patients with central NPSLE vs non-NPSLE patients	Anti-P may have a role in the pathogenesis of NPSLE	Low
Seth et al. (2020) [42]	Cross-sectional	SLE: 522, 488F, 34M, NPSLE: 167	28.5	1	Seizure, psychosis, mood disorder, stroke, demyelination	Anti-P	No significantly elevated biomarkers in NPSLE compared to non-NPSLE. Psychosis was significantly higher in patients with anti-P when compared to those without anti-P	Small sample size. Single-center study with a predominance of Tamil ethnicity; not longitudinal	Stroke was significantly higher in B2GP1 IgG compared to non-B2GP1; demyelination was significantly higher in LA + vs LA -; stroke had NL to high C3/C4 compared to those without strokes; Abs to MOG were present in patients with mood disorders compared to those without it	No individual biomarker was more prevalent in NPSLE compared to non-NPSLE; however, anti-P were significantly higher among certain NPSLE manifestations	Low
Tjensvoll et al. (2021) [43]	Cross-sectional	SLE: 67, PSS: 71	42	11	Cerebral dysfunction	Anti-NR2, anti-P	NfL levels increased in the CSF as the levels of anti-NR2 increased in the CSF; increased levels of anti-NR2 in CSF were associated with increased levels of NfL. No increase in NfL with increasing anti-P in SLE	Not longitudinal, no control, small sample size, difficult to obtain large CSF amounts; imaging could not detect small changes associated with anti-NR2; no adjustment for multiple testing was done	TWEAK levels were higher in PSS vs SLE patients; a positive correlation between NfL and anti-P in PSS; no association between NfL and S100B and TWEAK in SLE patients; intrathecal IgG increased with NfL in SLE patients	NfL in CSF is a marker of brain involvement in patients with SLE and reflects cognitive impairment in association with anti-NR2	Low
Yue et al. (2020) [44]	Cross-sectional, retrospective	78 patients: 9M, 69F	34	5.67	Cognitive dysfunction	Anti-NMDAR	Anti-NMDAR was higher in the NPSLE group compared to the non-NPSLE group	Single-center study; lacks data on accumulated prednisone doses	Anti-dsDNA was significantly higher in the non-NPSLE group than in the NPSLE group; C4 higher in the NPSLE group compared to the non-NPSLE group	The level of anti-NMDR is a predictor of cognitive dysfunction in SLE patients	Low
Abrol et al. (2021) [45]	Cross-sectional, retrospective	18 patients with lupus psychosis, 691 patients with SLE without psychosis, 709 total	26	2.9	Psychosis, depression, headache, seizure, anxiety, cognitive dysfunction, hypomania, visual disorder	Anti-NMDAR	A lack of NMDAR Abs was present on the surface of cells in NPSLE	Small NPSLE sample size; no data on psychosis symptoms prior to SLE diagnosis	Low lymphocytes and high anti-cardiolipin Abs in the non-NPSLE group compared to the NPSLE group; Anti-RNP were significantly higher in the NPSLE group compared to the non-NPSLE group	Lupus psychosis is a rare manifestation of SLE, and more information on biomarkers to identify Lupus psychosis is needed	Low
Buji et al. (2018) [46]	Cross-sectional	130 patients	Not stated	Not stated	Suicidal ideation, major depressive disorder, manic episodes, bipolar	Anti-NR2A	No association between NR2A and suicidal ideation in patients with SLE; heterozygous NR2A in combination with a higher WSAS score (for functional impairment) was associated with increased suicidal ideation	Potential underreporting of suicidal ideation; small sample size; not longitudinal	Pts who were unmarried, had a lifetime mood dysorder, currently depressed, had a previous suicide attempt, or scored high on the PHQ9, WSAS, or SRRS were more inclined to have suicidal ideation	NR2A alone is not associated with suicidal ideation, but there is a potential correlation between NR2A and functional impairment, increasing suicidal ideation in SLE patients	Low

Discussion

Overall, the reviewed evidence suggests anti-NR2/anti-NMDAR antibodies are primarily associated with neuropsychiatric manifestations, whereas anti-ribosomal P antibodies demonstrate stronger associations with psychiatric symptom burden and disease severity. These findings underscore the complexity and heterogeneity of neuropsychiatric involvement in SLE and highlight the limitations of autoantibodies as standalone biomarkers for predicting cognitive dysfunction.

Results showed anti-NR2/anti-NMDAR antibodies were associated with neuropsychiatric manifestations, including seizures, fibromyalgia, acute confusional states, mood disorders, and fatigue. Higher disease activity in patients with active NPSLE was independently associated with elevated levels of anti-NR2A and anti-NR2B antibodies [19-21]. These findings align with a proposed pathogenic mechanism in which anti-dsDNA antibodies cross-react with NMDA receptor subunits, with pathogenic effects facilitated by disruption of the blood-brain barrier during pro-inflammatory states, resulting in excitotoxic neuronal injury [10,11]. Additionally, elevations in antibodies were broadly linked to active disease states [21]. Together, these results suggest that anti-NR2 antibodies indicate inflammatory neuropsychiatric activity and symptom severity, but it is unclear whether they directly reflect the amount of neuronal damage. Thus, while anti-NR2 antibodies can function as markers for inflammatory neuropsychiatric activity, they are insufficient indicators for the degree of neuronal damage.

In contrast, serum anti-NR2 levels were not correlated with performance on standardized neuropsychological testing in long-standing SLE [25]. Cross-sectional analysis also did not correlate anti-NMDA antibodies with MoCA scores [26]. Longitudinal data further demonstrated that anti-NMDAR antibodies did not predict cognitive decline or damage accrual over time [27]. These findings suggest that cognitive impairment in SLE likely reflects multifactorial contributors beyond isolated antibody-mediated excitotoxicity.

Across the reviewed studies, anti-ribosomal P antibodies showed a strong association with psychiatric manifestations and overall disease severity rather than isolated cognitive impairment. These results are consistent with prior literature, indicating anti-ribosomal P antibodies against ribosomal phosphoproteins are associated with an increased prevalence of neuropsychiatric involvement, including mood disorders and psychosis [8,13]. Multiple cohorts demonstrated significant associations with anti-P positivity and symptoms of depression, anxiety, stroke, and broader neuropsychiatric involvement [28,29,31]. These findings were further supported by neuroimaging showing clinical correlation through functional MRI alterations in regions involved in emotional regulation in anti-P-positive patients experiencing depressive symptoms [32].

In addition to the psychiatric symptom burden, anti-ribosomal P antibodies were associated with increased disease activity - the more severe clinical phenotype indicated by higher SLEDAI-2k scores and increased early and long-term mortality in NPSLE [33,34]. These findings demonstrate that the presence of anti-ribosomal P antibodies can be useful in identifying patients with systemically active and clinically severe disease.

As with anti-NR2 antibody-positive patients, there was limited evidence of a direct relationship between anti-ribosomal P antibody positivity and cognitive impairment. Additionally, one cross-sectional cohort showed no significant association between anti-P antibodies and overall NPSLE status or major organ involvement [35]. Thus, while anti-ribosomal P antibodies appear robustly linked to psychiatric symptom burden and disease activity, their role in isolated cognitive impairment is still unclear.

The reviewed literature suggests that anti-NR2/anti-NMDAR antibodies and anti-ribosomal P antibodies have independent but partially overlapping neuropsychiatric patterns. Although neither antibody shows a consistent association with global cognitive impairment across heterogeneous SLE populations [14,25], they appear to correlate with different domains of neuropsychiatric involvement. Anti-NR2/anti-NMDAR antibodies have been linked to neuropsychiatric manifestations and generalized fatigue, whereas anti-ribosomal P antibodies were associated with psychiatric symptom burden and disease severity [20,21,23,30,33]. Identifying these antibody profiles remains clinically relevant because neuropsychiatric symptoms in SLE are often nonspecific and difficult to attribute to lupus versus other causes. As a result, antibody markers may provide supportive biological evidence for underlying disease mechanisms, help stratify patients into biologically meaningful subgroups, and potentially aid in earlier recognition or monitoring of neuropsychiatric disease activity even when overt clinical phenotypes are heterogeneous or evolving.

From a clinical standpoint, the findings suggest autoantibody profiles may be useful for phenotypic stratification and risk assessment rather than as a diagnostic marker for isolated cognitive impairment in SLE. The association of anti-NR2/anti-NMDAR antibodies with neuropsychiatric manifestations suggests possible utility in identifying active or inflammatory neuropsychiatric involvement, especially in periods of increased disease activity [20-24]. Similarly, anti-ribosomal P antibodies serve a useful role in identifying the psychiatric symptom burden and the severity of disease [30,31,33,34].

Additionally, the absence of a consistent association between either antibody or objective cognitive impairment across multiple cohorts further emphasizes the need for a comprehensive clinical evaluation, rather than relying on serological markers when assessing cognitive complaints in SLE [14,25,26]. Given the complexity and heterogeneity of NPSLE, a multimodal clinical approach is required, integrating neuropsychiatric assessments, diagnostic imaging, and individualized monitoring for effective diagnosis and management of the disease.

Interpretation of data may have been limited by the heterogeneity across study designs, antibody assays, patient populations, and neuropsychiatric assessment tools. The variability in antibody measurement techniques and thresholds limits the ability for direct comparison across cohorts as well as generalizability.

## Conclusions

The incorporation of autoantibody profiling into the evaluation of NPSLE offers a structured framework to characterize disease heterogeneity and guide risk stratification more effectively. Across the studies reviewed, anti-NR2/anti-NMDAR antibodies were most consistently associated with neuropsychiatric manifestations and generalized fatigue. In contrast, anti-ribosomal P antibodies were more strongly linked to psychiatric symptom burden and greater overall disease activity. These patterns reinforce the concept that distinct antibody profiles may reflect different inflammatory and neurobehavioral phenotypes within NPSLE rather than a single uniform process. However, neither antibody demonstrated a consistent or independent relationship with global cognitive impairment. This variability suggests that antibody utility may depend on clinical context, disease activity, and patient-specific factors, rather than serving as standalone predictive biomarkers. Further studies are necessary to clarify how these antibodies can be integrated into multimodal diagnostic strategies that combine serologic markers, cerebrospinal fluid analysis, imaging, and formal neuropsychological testing. These findings highlight both the potential role of antibody profiling in phenotypic stratification and the importance of cautious interpretation when assessing cognitive complaints in patients with SLE.
